# Emerging COVID-19 reinfection four months after primary SARS-CoV-2 infection

**DOI:** 10.1007/s10354-021-00813-1

**Published:** 2021-02-08

**Authors:** Helmut J. F. Salzer, Matthias Neuböck, Sven Heldt, Isabella Haug, Christian Paar, Bernd Lamprecht

**Affiliations:** 1 Department of Pulmonology, Kepler University Hospital, Linz, Austria; 2 Institute of Laboratory Medicine, Kepler University Hospital, Linz, Austria

## To the editor

In the last few months, several cases of ominous coronavirus disease 2019 (COVID-19) reinfections have been reported (Table 1). However, there is a scientific controversy whether reinfections can occur just a few months after the first infection and if so, what it means for the fight against the COVID-19 pandemic.

**Table 1:** Clinical characteristics of symptomatic COVID-19 reinfections having a negative SARS-CoV‑2 PCR between the first and the second infection and/or a phylogenetic analysis

Country	Sex	Age (years)	Comorbidities	1st infection	2nd infection	Interval between 1st and 2nd infection	Negative SARS-CoV-2 PCR between 1st and 2nd infection	Phylogenetic analysis	Reference
Israel	Female	20	None	Mild^a^	Asymptomatic	112 days	Yes	No	[5]
Ecuador	Male	46	N/A	Mild	Mild	63 days	N/A	Yes	[6]
USA	Male	82	Parkinson’s disease, diabetes, chronic kidney disease, hypertension	Severe^b^	Severe	55 days	Yes	No	[7]
Hong-Kong	Male	33	N/A	Mild	Asymptomatic	142 days	Yes	Yes	[4]
USA	Male	25	None	Mild	Mild	48 days	Yes	Yes	[8]
Belgium	Female	51	Asthma (inhaled corticosteroids)	Mild	Mild	93 days	No	Yes	[9]
The Netherlands	Female	89	Waldenström’s macroglobulinemia	Mild	Moderate^c^	59 days	No	Yes	[10]
USA	N/A	N/A	Emphysema, home oxygen, hypertension	Moderate	Moderate	144 days	Yes	Yes	[11]
USA	Male	42	N/A	Mild	Mild	51 days	No	Yes	[12]
Brazil	1 × Female, 2 × Male	40, 67, 47	Asthma, ancylosing spondylitis, obesity, OSAS, none	Mild	Mild to severe	54, 56, 70 days	Yes	No	[13]
Austria	Male	95	Dementia, hypertension, total thyroidectomy	Mild	Severe	124 days	Yes	No	–

*COVID-19* coronavirus disease 2019, *SARS-CoV‑2* severe acute respiratory syndrome coronavirus 2, *PCR* polymerase chain reaction, *N/A* not available, *OSAS* obstructive sleep apnea syndrome

^a^Symptomatic with absence of hypoxia

^b^Critical ill requiring non-invasive or invasive ventilation and/or death related to COVID-19

^c^Symptomatic requiring additional oxygen

On October 27, 2020, a 95-year-old man was re-admitted from his retirement home to Kepler University Hospital in Linz, Austria with new onset dyspnea and fever. Four months before, he had been discharged after 2 weeks of hospitalization due to mild COVID-19 characterized by fever and leukopenia, but absence of viral pneumonia and hypoxia. For virological confirmation a severe acute respiratory syndrome coronavirus 2 (SARS-CoV-2) reverse transcription polymerase chain reaction (RT-PCR) was performed showing positive test results on June 27 with a cycle threshold (Ct) value of 32.2 and on July 2, 2020 with a Ct value of 37.7 (cobas 6800 SARS-CoV‑2 test, Roche, Molecular Systems, Branchburg, NJ, USA). Thereafter, the patient tested negative for SARS-CoV‑2 on several occasions including at discharge from hospitalization on July 6 and 7 as well as on September 25 and on October 1, 2020. The patient had a medical history of dementia, arterial hypertension and total thyroidectomy.

Referring to local COVID-19 infection precaution regulation the patient was directly isolated in the emergency room and he was again tested for SARS-CoV‑2 on October 27, 2020. Meanwhile vital parameters were taken showing a reduced oxygen saturation of 89% on room air and an elevated body temperature of 38.4 °C. Auscultation of the lung revealed no pathological abnormalities, while laboratory test results showed mild leukopenia with 3.18 G/L (reference value 3.9–8.8 G/L) with a decreased lymphocyte count of 0.64 G/L (reference value 1.00–4.00 G/L) and a thrombocytopenia with 126 G/L (reference value 151–400 G/L), respectively. Other laboratory values and urine test results were unremarkable.

Two hours later the patient was again tested positive for SARS-CoV‑2 with a Ct value of 12.8 in the RT-PCR (Cepheid Xpert Xpress SARS-CoV-2 point-of-care test, Sunnyvale, CA, USA). Another oropharyngeal swab was taken confirming the positive SARS-CoV‑2 RT-PCR test result with a Ct value of 14.5 using a different platform (cobas 6800 SARS-CoV‑2 test, Roche, Molecular Systems, Branchburg, NJ, USA).

Despite primary SARS-CoV‑2 infection the patient this time required additional oxygen and had viral pneumonia on chest X‑ray. Furthermore, the patient received low molecular weight heparin with enoxaparin 4000 I.E. subcutaneously once daily for prophylaxis of venous thromboembolism and paracetamol 1000 mg intravenously as antipyretic treatment. Antiviral treatment was not administered due to drug shortage of remdesivir in Upper Austria at this time. We did not give dexamethasone at admission because the patient was not critically ill, he had no laboratory findings of hyperinflammation and was in the early phase of viral infection. Over the next few days the patients’ respiratory condition deteriorated continuously consistent with a severe course of COVID-19. Finally, the patient deceased 6 days after admission.

Taken this together a COVID-19 reinfection seems to be plausible in our patient 124 days after primary SARS-CoV‑2 infection, although a recently published clinical meta-analysis including 15 single or cumulative case reports did not find any clinical reinfection after a 70-day period following first infection [1]. These findings are supported by animal studies demonstrating protection against reinfection in rhesus macaques after primary exposure to SARS-CoV‑2 [2, 3].

Nevertheless, the first and the second COVID-19 episode in our patient were characterized by clinical symptoms, typical laboratory findings including leukopenia and thrombocytopenia as well as repeated virological confirmation of SARS-CoV‑2 infection, while he had no symptoms and he tested negative on several occasions in between. To KK‑W et al. also reported a reinfection in a 33-year-old man 142 days after first infection. Whole genome sequencing confirmed that both COVID-19 episodes were caused by phylogenetically diverse SARS-CoV‑2 strains, which supports our clinical observation of reinfection instead of persistent viral shedding [4]. Questions remain, for example, why this patient acquired a COVID-19 reinfection, while immunity against the virus is probable, at least in the short term, since SARS-CoV‑2 reinfections are only reported occasionally despite the high COVID-19 prevalence worldwide. Explanations could be an infection with a different SARS-CoV‑2 strain or an age-related impaired immune response. Unfortunately we were not able to perform a comparison of whole genome sequencing data due to missing sample material of the first episode of infection.

We want to draw attention to this emerging aspect in the COVID-19 pandemic since reinfections will certainly influence our future scientific, clinical, social and economic response to COVID-19 pandemic. It will raise considerable questions on innate and adaptive immune response, on herd immunity and on vaccine development.
